# How to Capitalise on Media Communications to Promote Mental Healthcare for Left-Behind Children in China

**DOI:** 10.3389/ijph.2023.1606023

**Published:** 2023-07-03

**Authors:** Jason Hung

**Affiliations:** ^1^ Department of Sociology, The University of Cambridge, Cambridge, United Kingdom; ^2^ Institute of Sociology, Academia Sinica, Taipei, Taiwan

**Keywords:** left-behind children, China, media, mental health, policy

**The IJPH series “Young Researcher Editorial” is a training project of the Swiss School of Public Health.**

In China, where there is a notable urban-rural divide and rural areas are underdeveloped, parents leave children behind in their villages and relocate to cities or towns to seek better career opportunities and higher income. There are 69.7 million left-behind children (LBC) in China [1]. LBC are particularly vulnerable to mental health issues like depression, anxiety, and developing anti-social personalities [2]. The rural mental healthcare infrastructure may be insufficient to provide necessary psychological services, most of which are available only in cities. Rural children, including LBC, are usually underserved [3], but media communications can be optimised to treat and prevent LBC from developing mental health problems.

The Chinese government has recently implemented a range of targeted policies, including the “Double Support Plan” launched in 2016. This plan aims to improve the physical and mental health of LBC by providing them with psychological counselling services. Under the scheme, LBC are prioritised above other children to access psychological counselling services. The initiative requires rural primary and secondary schools to dispatch social workers to rural communities to provide counselling services [4]. If utilized to maximum effect, the existing mental healthcare system and media communications infrastructure can effectively support delivery of appropriate healthcare to LBC. There are three main obstacles to delivering care.

First, local rural governments have consistently failed to disseminate mental healthcare-related information to the local populations on a regular basis via localised channels of traditional and social media. The resulting lack of information keeps rural usage rates of mental healthcare facilities low even when they are available. Even in villages with available facilities, primary caregivers of LBC do not know how to access to local mental healthcare services including mental health screening, psychological counselling services, and mental health treatment [5]. To make this information widely available, local rural governments should advertise on local television channels and mobile apps like WeChat (a Chinese version of WhatsApp with additional digital and social media functions) so those in need know what mental healthcare services are available and how to access them. Local governments must also build more mental health clinics and train and provide financial incentives to mental health professionals who work in villages.

Second, China does not provide comprehensive mental health education for school teachers and children’s caregivers [6], who are usually the first points of contact for psychologically vulnerable LBC. Teachers and caregivers must be educated and trained to identify early warning signs of psychological problems in LBC so they can intervene early. They must learn to advise students to mitigate the psychological and emotional effects of being left behind. Teachers and caregivers can use mobile apps to deliver care and show warmth to LBC and express concern about their wellbeing, since LBC are in need of extra support. Local rural government and mental healthcare representatives can also use mobile apps to regularly disseminate mental health-related information, educating the public about possible psychological upheaval in LBC. Local rural governments should collaborate with available mental healthcare facilities; health professionals from these facilities should regularly disseminate information on localised traditional and social media platforms to educate teachers and caregivers about how they should react if LBC display signs of mental health problems.

Third, most urban and rural Chinese own smartphones and local rural policymakers should capitalise on their popularity to promote mental healthcare services. If trustworthy role models, including their own guardians, parents, teachers and health professionals, can use these apps to provide care, warmth, support, professional advice, and supervision to LBC, that should mitigate the vulnerability of LBC and satisfy the psychological and emotional needs of these children. Smartphones and apps can deliver adult support and advice to any location and any time. While not an ideal solution, they can increase the social inclusion of LBC and maintain relationships between parents, teachers, mental health professionals, and children, connecting them to families and communities.
